# Dietary Guanidinoacetic Acid Improves Meat Tenderness and Antioxidant Capacity in Rabbits via Modulating Muscle Fiber Characteristics and Fat Metabolism

**DOI:** 10.3390/ani16121827

**Published:** 2026-06-12

**Authors:** Yanhui Liang, Xi Chen, Xiaoyu Fan, Yingmei Zhang, Shengnan Wang, Xiaojia Wu, Yingle Wei, Changmao Wei, Yichen Lin, Qinghua Liu, Changchuan Ye

**Affiliations:** 1 Fujian Provincial University Engineering Research Center for Animal Breeding and Sustainable Production, College of Animal Sciences, Fujian Agriculture and Forestry University, Fuzhou 350002, China; 13599873290@163.com (Y.L.); 17852396033@163.com (X.F.); 13618540600@163.com (Y.Z.); 18748795318@163.com (S.W.); wxj15396080092@outlook.com (X.W.); 18867054246@163.com (Y.W.); 15117816467@163.com (C.W.); a15960022520@163.com (Y.L.); 2 Fujian Academy of Agricultural Sciences, Fuzhou 350013, China; chenximelon@126.com

**Keywords:** guanidinoacetic acid, rabbits, production performance, antioxidant capacity, muscle fiber properties, fatty acid metabolism

## Abstract

As the direct biosynthetic precursor of creatine, guanidinoacetic acid (GAA) exerts a pivotal regulatory role in energy homeostasis and protein metabolism. Rabbit meat has garnered increasing global recognition as a healthy food source, characterized by its outstanding high-protein and low-fat nutritional profile. Accordingly, the optimization of rabbit meat quality has attracted growing attention from both consumers and animal production practitioners. In the present study, we evaluated the impacts of dietary GAA supplementation on meat quality traits, in vivo antioxidant capacity, muscle fiber characteristics, and fatty acid metabolism in New Zealand white rabbits. A total of 960 male New Zealand white rabbits were assigned to two age groups: 40-day-old group and 60-day-old group (40 ± 2 days, 1.19 ± 0.09 kg; 60 ± 2 days, 1.82 ± 0.15 kg). Within each age group, rabbits were randomly allocated to a control diet or a diet supplemented with 100 mg/kg GAA (CON-40, GAA-40, CON-60, GAA-60). After a 45-day feeding period, two-way ANOVA revealed that GAA supplementation significantly reduced shear force (*p* < 0.01, diet main effect) and muscle fiber density (*p* < 0.01, diet main effect), with an age-dependent effect on shear force (age × diet interaction, *p* < 0.05). Moreover, GAA enhanced systemic antioxidant capacity, as indicated by increased serum superoxide dismutase (SOD) activity (*p* < 0.01) and total antioxidant capacity (T-AOC) (*p* < 0.05), while no significant effect on malondialdehyde (MDA) was detected under the current experimental conditions. GAA also regulated the expression of lipid metabolism-related genes (*FAS*, *HSL*, *ACC*) in intramuscular and perirenal fat, indicating its regulatory effect on fatty acid metabolism. In conclusion, dietary GAA supplementation improves rabbit meat tenderness and antioxidant capacity, with no negative effects on growth performance. These findings confirm that GAA has the potential to serve as a nutritional strategy to improve rabbit meat quality, supporting the development of rabbit meat as a functional food for human consumption.

## Simple Summary

Rabbit meat is widely favored as a healthy protein source. Accordingly, this study was conducted to investigate the effects of dietary guanidinoacetic acid supplementation on meat quality, antioxidant capacity, myofiber characteristics and fatty acid metabolism in New Zealand white rabbits, aiming to provide a novel nutritional strategy for improving rabbit meat quality. Our findings indicate that dietary guanidinoacetic acid supplementation enhances meat tenderness and antioxidant capacity without compromising growth performance, highlighting guanidinoacetic acid as a promising feed additive for improving the quality of rabbit meat, in line with the growing consumer demand for healthier and more sustainably produced animal products.

## 1. Introduction

The rising global demand for high-quality animal protein, coupled with challenges in sustainable food production, has intensified efforts to boost the nutritional value and eating quality of meat products [1]. In this context, rabbit meat has gained increasing attention as a healthy and sustainable protein source. It is characterized by high protein content, low fat, low cholesterol, and favorable amino acid and fatty acid profiles, making it particularly suitable for health-conscious consumers and individuals with dietary restrictions [2]. Consequently, improving the quality of rabbit meat has become a priority for both producers and researchers. However, challenges remain in consistently achieving optimal meat quality in commercial rabbit production, particularly in terms of tenderness, water-holding capacity, and oxidative stability, emphasizing the need for effective nutritional strategies.

Guanidinoacetic acid (GAA), the immediate metabolic precursor of creatine, is endogenously synthesized from arginine and glycine and has been widely recognized to play a pivotal role in cellular energy homeostasis [3,4,5]. As a feed additive, GAA holds multiple advantages over direct creatine supplementation, including greater chemical stability, lower production cost, and higher bioavailability [6,7]. Upon absorption, GAA is methylated to form creatine, which is then phosphorylated to phosphocreatine. Phosphocreatine serves as a key energy buffer, helping to maintain ATP levels in tissues characterized by high and dynamically fluctuating energy requirements, including skeletal muscle [5,8]. Beyond its well-established role in energy metabolism, emerging evidence suggests that GAA may exert broader physiological effects, including modulation of muscle fiber characteristics, antioxidant defense, and lipid metabolism.

In recent years, the regulatory effects of dietary GAA inclusion on meat quality have been investigated across various livestock species. In ruminants, GAA supplementation improved carcass quality by reducing excessive fat deposition in subcutaneous and visceral adipose tissues [8,9,10]. In Hu sheep, dietary GAA increased muscle shear force values and fiber diameter, with a concurrent decline observed in drip loss percentage and the density of muscle fibers [11]. In pigs, dietary GAA at 0.045% enhanced the quality characteristics of pork by altering the structural traits of muscle fibers and reducing mandibular fat accumulation [12], while 0.12% GAA fed pre-slaughter period improved lean meat percentage and decreased backfat deposition thickness [13]. In poultry, GAA supplementation improved broiler performance without adversely affecting meat quality [14,15]. Collectively, these findings indicate that GAA can modulate muscle fiber properties and lipid metabolism, thereby influencing meat quality across multiple species.

Although the efficacy of GAA has been well-documented in various livestock and poultry species, research reports on its application in rabbit production are currently very limited, and its impact on rabbit meat quality has not been systematically evaluated. Therefore, we conducted this study to investigate the influences of dietary supplemented GAA on meat quality traits, antioxidant status, muscle fiber phenotypic characteristics, and fatty acid metabolism in rabbits. Being one of the most globally prevalent meat rabbit breeds, the New Zealand rabbit was chosen for this experiment as an ideal experimental model. At the same time, given the varying preferences of consumers regarding rabbit body size, rabbits at two different ages were used in this trial to provide data supporting the optimal period for commercial supplementation of guanidinoacetic acid. The findings are expected to provide a scientific basis for using GAA as a nutritional intervention strategy to enhance meat quality traits of rabbit meat as a functional food for human intake.

## 2. Materials and Methods

### 2.1. Moral Statement

The study was approved by the Fujian Agriculture and Forestry University Animal Care and Use Committee (Approval ID: PZCASFAFU24122) on 15 January 2024.

### 2.2. Experimental Materials

A total of 960 healthy male New Zealand white rabbits were selected from Fujian Chunlong Agriculture and Animal Husbandry Technology Co., Ltd. (Fuzhou, China). The animals comprised two age groups: 480 rabbits aged 40 ± 2 days (body weight 1.24 ± 0.09 kg) and 480 rabbits aged 60 ± 2 days (body weight 1.87 ± 0.15 kg). Guanidinoacetic acid (GAA, purity ≥ 99%) used in the trial was supplied by Beijing Gendone Biotechnology Co., Ltd. (Beijing, China).

### 2.3. Housing Conditions and Feeding Management

The experiment was conducted at the Fujian Chunlong Agriculture and Animal Husbandry Technology Co., Ltd. (Fuzhou, China). A randomized block design was employed in this study. The experimental rabbits were reared in individual pens, with 24 rabbits allocated to each pen. Prior to the trial, the rabbit house, flooring, pens, feeders, and drinking systems were thoroughly cleaned and disinfected. All rabbits were ear-tagged and vaccinated before the start of the study.

During the experimental period, weekly disinfection and cleaning of the housing and pens were performed routinely. Feeders were cleaned daily, and drinking equipment along with cooling pads were inspected to ensure proper function. The animals were housed in a clean and quiet environment, with ambient temperature maintained at 23–28 °C and relative humidity at approximately 80%. Feeding was carried out at 7:00, 15:00, and 20:00 daily. Over the entire experimental trial, rabbits had unlimited access to feed and fresh water. All other husbandry practices, including immunization procedures, followed standard operating protocols.

The basal diet was formulated according to NRC (1994) guidelines, and the composition and nutritional levels of the basal diets of the test rabbits are shown in Table 1.

### 2.4. Experimental Design

**Figure 1:**
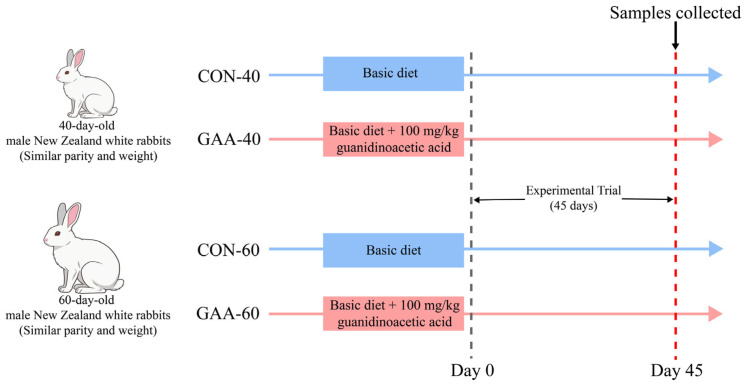
The schematic diagram of experimental design in this study.

As illustrated in Figure 1, weaned male rabbits were selected and divided them into two age groups: 40-day-old group and 60-day-old group. Within each age group, rabbits were randomly divided into two dietary treatments: control groups (CON-40, CON-60) and groups supplemented with 100 mg/kg GAA (GAA-40, GAA-60). Each treatment group consisted of 240 rabbits, which were assigned to 10 experimental replicates. Each replicate, comprising 24 rabbits, was housed in a single dedicated pen. The experiment consisted of a 7-day adaptation period followed by a 45-day formal feeding period. In a preliminary trial, graded levels of 0, 50, 100, and 150 mg/kg GAA were added to the basal diet. The inclusion of 100 mg/kg GAA showed the most pronounced effects in meat quality and was therefore selected for the formal experiment. The data of preliminary trial was shown in Table S1. During the formal trial, the control groups (CON-40 and CON-60) received the basal diet, whereas the GAA-supplemented groups (GAA-40 and GAA-60) were provided with basal diet fortified with 100 mg/kg GAA. The supplement was first mixed into a premix and then uniformly blended with other ingredients before pelleting. Throughout the study, all animals were raised under standardized management practices to ensure consistent experimental conditions and reliable data collection.

### 2.5. Sample Collection

Feed intake of each replicate (pen) was monitored throughout the experimental period. Body weight of all rabbits was measured at the start and termination of the trial for the calculation of average daily feed intake (ADFI), average daily gain (ADG), and feed-to-gain ratio (F/G). Given the adoption of a group housing system in this study, each experimental replicate (i.e., each pen) was considered an individual experimental unit for subsequent statistical analysis. At the end of the 45-day feeding period, five rabbits per group were randomly selected for slaughter (one rabbit from every two replicates). All blood collection and slaughter were performed at 9 a.m. on the same day. Serum was prepared by collecting blood from the jugular vein, incubating samples at 4 °C for 2 h, followed by centrifugation at 3000× *g* for 15 min, and then stored at −20 °C for later biochemical and antioxidant analyses. Rabbits were stunned by electrical stunning (120 V, 50 Hz, 3 s) to minimize pain, followed by rapid and complete exsanguination via carotid artery and jugular vein incision for 5 min. Following slaughter, animals were processed by removing the skin, head, feet, tail and internal organs, while leaf fat and kidneys were kept intact. The resultant body mass was recorded as carcass weight, and dressing percentage was determined by dividing carcass weight by live weight before slaughter. The heart, liver, spleen, lungs, and kidneys were removed, surrounding fat was trimmed off, and blood stains were blotted with filter paper. They were then accurately weighed for the calculation of organ indices. Samples of the longissimus thoracis et lumborum were collected. Muscle specimens intended for histological analysis were fixed immediately; liquid nitrogen was used for snap-freezing all other tissue samples, which were thereafter maintained at −80 °C until subsequent analysis.

Dressing percentage = carcass weight/live weight before slaughter × 100%

Organ index = organ weight (g)/live weight before slaughter (kg) × 100%

### 2.6. Meat Quality and Histological Analysis

After slaughter, samples of the longissimus thoracis et lumborum were collected. Surrounding fat and connective tissues were removed for the assessment of pH values, drip loss, and shear force. Measurements of pH were conducted via a portable pH meter (PHSJ-3F, Shanghai, China). Before measurement, a two-point calibration was performed using pH 4.00 and 7.00 standard buffers, and automatic temperature compensation was enabled throughout. After each group of samples was measured, the calibration was rechecked. Specifically, the pH meter probe was inserted into the longissimus thoracis et lumborum at a depth of 3 mm, and three individual positions were selected for pH measurement, and their readings were averaged for subsequent analysis. Drip loss was determined by taking a 3 g sample of the longissimus thoracis et lumborum, hooking it with a wire, placing it into a sealed bag without contacting the bag, hanging it in a refrigerator at 4 °C for 24 h, and then weighing the sample [16]. For shear force measurement, samples of the longissimus thoracis et lumborum were taken and samples were trimmed of fat and connective tissue. After being placed at room temperature for 1 h, a constant-temperature water bath set at 80 °C was used to heat the meat specimens until their core temperature attained 70 °C. They were then removed and cooled to 20 °C. Along the direction of the muscle fibers, the samples were trimmed into 1 cm × 1 cm rectangular meat strips. A C-LM3B digital tenderness meter was used to shear the samples perpendicular to the fiber direction, and the shear force values were recorded. Every specimen was detected three independent times, and the calculated mean served as the ultimate result. Intramuscular fat content was determined by petroleum ether extraction.

Drip loss (%) = (meat weight before storage − meat weight after 24 h)/meat weight before storage × 100%

For histological analysis, longissimus thoracis et lumborum samples were collected immediately after slaughter and fixed in universal tissue fixative, ensuring complete submersion. After fixation, samples were dehydrated, trimmed, embedded, sectioned, and stained following standard procedures. After staining, a Nikon Eclipse Ci-L upright brightfield microscope was used to select the target area of the tissue for imaging at 200× magnification. Subsequently, Image-Pro Plus 6.0 analysis software was utilized to measure muscle fiber diameters in a millimeter scale. For each section, five individual muscle fibers were assessed, and their average diameter was computed. The number of muscle fibers in each section was counted, and the corresponding total area of the muscle fibers was measured, from which the muscle fiber density was calculated.

Muscle fiber density = muscle fiber count/total muscle fiber area

### 2.7. Serum Biochemical Indexes and Antioxidant Capacity

Among the five serum samples per group, three were randomly selected for the measurement for aspartate aminotransferase (AST), alanine aminotransferase (ALT), high-density lipoprotein (HDL), and low-density lipoprotein (LDL). Commercial kits (Hunan Yonghe Sunshine Technology Co., Ltd., Hunan, China) were used for the measurements.

Antioxidant indices including superoxide dismutase (SOD), catalase (CAT), malondialdehyde (MDA), and total antioxidant capacity (T-AOC) were detected using serum samples. Commercial kits (Shanghai Liquid Quality Assay Technology Co., Ltd., Shanghai, China) were used to measure the antioxidant capacity following the kit manufacturer’s specifications.

### 2.8. Real-Time Quantitative PCR of Gene Expression

Among the five rabbits slaughtered per group, three were randomly selected for analyses of gene expression. Samples of longissimus thoracis et lumborum and perirenal fat were collected, placed in 5 mL centrifuge tubes, immediately snap-frozen in liquid nitrogen, and stored at −80 °C until analysis.

NucleoZol reagent (Gene, Düren, Germany) was applied to isolate total RNA, and a reverse transcription kit (Servicebio, G3330-100, Wuhan, China) was used for cDNA synthesis. All qPCR reactions were run on the ABI 7300 system (Applied Biosystems, Foster City, CA, USA). All reactions were carried out in a final volume of 20 μL, consisting of 10.0 μL of 2× SYBR qPCR Master Mix, 1.0 μL each of 10 μM forward and reverse primers, 1.0 μL of cDNA template, and RNase-free double-distilled water to bring the total volume to 20 μL. The thermal cycling program was set as follows: an initial pre-denaturation step at 95 °C for 5 min with 1 cycle; followed by 40 amplification cycles, each comprising denaturation at 95 °C for 10 s, annealing at 60 °C for 30 s, and extension at 72 °C for 30 s; after amplification, a melt curve analysis was performed to validate the specificity of the amplification products, with the program set to 1 cycle of 95 °C for 15 s, 60 °C for 60 s, and a final step at 95 °C for 15 s. Primer sequences for fatty acid synthase (*FAS*), hormone-sensitive lipase (*HSL*), acetyl-CoA carboxylase (*ACC*), and the endogenous reference gene β-actin (*ACTIN*) are presented in Table 2. All reactions were performed in triplicate. The 2^−ΔΔCT^ method was employed to determine the relative expression of target genes after normalization to *ACTIN* [17].

### 2.9. Statistical Analysis

All statistical analyses were carried out via SPSS 27 software and GraphPad Prism (9.0.0). The effects of diet (supplemented with 100 mg/L GAA), age, and their interaction were analyzed by two-way analysis of variance using the general linear model (GLM) procedure. Results are shown as mean ± SEM, and statistical significance was defined at *p* < 0.05.

Notably, as this study was a commercial trial, the sample size was relatively limited due to cost considerations, which may have had some impact on the results. In future studies, we will use a larger sample size and a more comprehensive experimental design to validate these preliminary findings.

## 3. Results

### 3.1. Growth Performance and Carcass Traits

As can be seen from Table 3, the 60-day-old group exhibited significantly greater values in initial body weight, final body weight, average daily feed intake, F/G, live weight before slaughter, and carcass weight when compared with the 40-day-old group (*p*_(age)_ < 0.01), whereas average daily gain and dressing percentage were not significantly affected (*p*_(age)_ > 0.05). The addition of GAA to the diet exerted no effect on growth performance or carcass traits (*p*_(diet)_ > 0.05), and no age × diet interaction was detected for these variables (*p*_(age×diet)_ > 0.05).

### 3.2. Organ Indices

**Figure 2:**
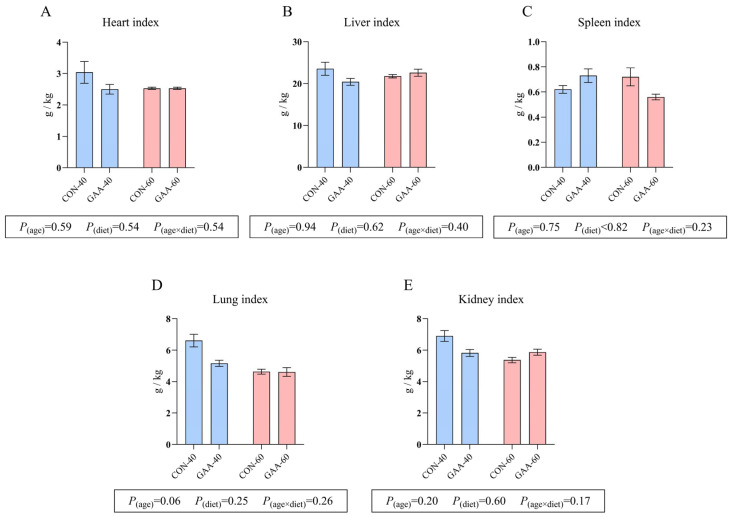
Effect of addition of GAA on the development of organs in rabbits ((**A**–**E**): organ index of heart, liver, spleen, lung and kidney). The error bar represents the standard error of means (SEM). *n* = 5.

As shown in Figure 2, neither age nor dietary supplementation with GAA significantly affected the heart, liver, spleen, lung, or kidney indices (*p*_(age)_ > 0.05, *p*_(diet)_ > 0.05), and no age × diet interaction was observed for any of these organ indices (*p*_(age×diet)_ > 0.05).

### 3.3. Meat Quality and Muscle Fiber Characteristics

**Figure 3:**
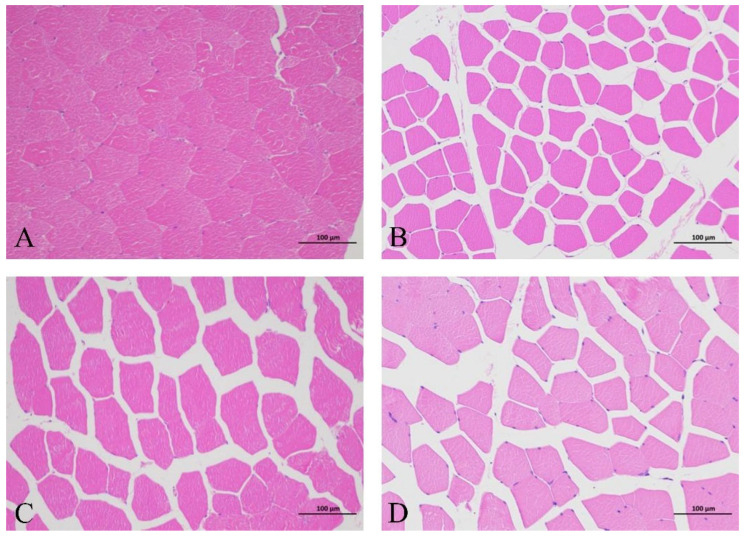
Microscopic images of the longissimus thoracis et lumborum cross-sections from the CON-40 (**A**), GAA-40 (**B**), CON-60 (**C**), and GAA-60 (**D**) groups. GAA was supplemented at 100 mg/kg in the GAA group.

Dietary GAA supplementation affected meat quality in an age-dependent manner (Table 4, Figure 3). pH_45min_ was significantly affected only by age (*p*_(age)_ < 0.01), declining markedly with increasing age, whereas dietary GAA and the age × diet interaction showed no significant effect (*p*_(diet)_ > 0.05, *p*_(age×diet)_ > 0.05). Drip loss was significantly influenced only by age (*p*_(age)_ < 0.05), increasing as age advanced, while GAA and the interaction were not significant (*p*_(diet)_ > 0.05, *p*_(age×diet)_ > 0.05). Shear force was significantly affected by both age and dietary GAA (*p*_(age)_ < 0.01, *p*_(diet)_ < 0.01), and their interaction was also significant (*p*_(age×diet)_ < 0.05); shear force increased markedly with age, whereas GAA supplementation significantly decreased it, with a greater reduction observed in 60-day-old group. No significant differences in muscle fiber diameter and total muscle fiber area were detected among age, GAA, or their interaction (*p*_(age)_ > 0.05, *p*_(diet)_ > 0.05, *p*_(age×diet)_ > 0.05). Muscle fiber density was significantly influenced by both age and dietary GAA (*p*_(age)_ < 0.01, *p*_(diet)_ < 0.01), without a significant interaction (*p*_(age×diet)_ > 0.05); density decreased markedly with increasing age, and dietary GAA significantly reduced it.

### 3.4. Serum Biochemical Parameters

**Figure 4:**
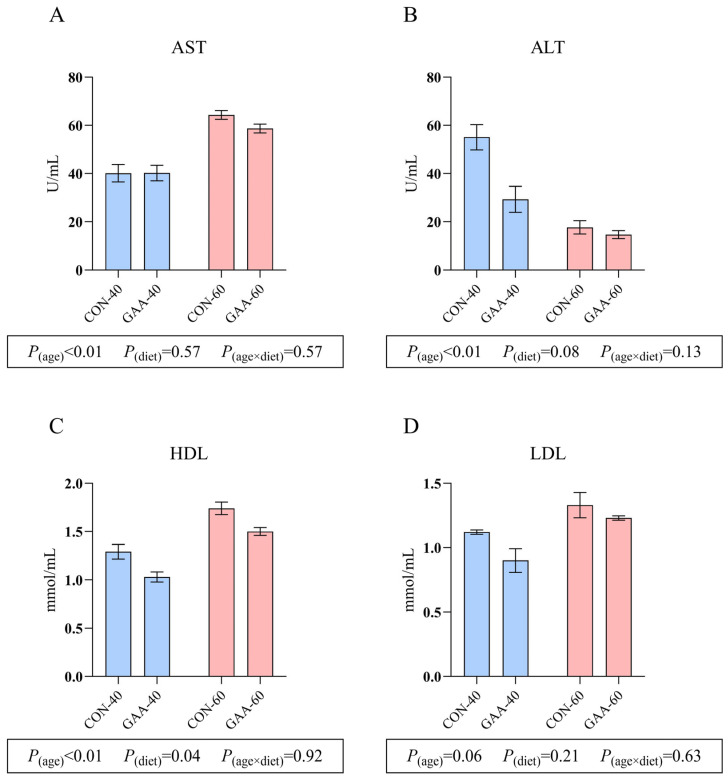
Serum biochemical parameters of rabbits fed diets with or without GAA. (**A**) AST, (**B**) ALT, (**C**) HDL, (**D**) LDL. The error bar represents the standard error of means (SEM). *n* = 3. AST, aspartate aminotransferase; ALT, alanine aminotransferase; HDL, high-density lipoprotein cholesterol; LDL, low-density lipoprotein cholesterol.

As shown in Figure 4, AST activity was significantly influenced only by age (*p*_(age)_ < 0.01), increasing with age, whereas dietary GAA and the age × diet interaction had no significant effect (*p*_(diet)_ > 0.05, *p*_(age×diet)_ > 0.05). ALT activity was also significantly affected only by age (*p*_(age)_ < 0.01), but it decreased with age, with no significant effects of GAA or the interaction (*p*_(diet)_ > 0.05, *p*_(age×diet)_ > 0.05). HDL level was significantly influenced by age (*p*_(age)_ < 0.01) and dietary GAA (*p*_(diet)_ < 0.05), without a significant age × diet interaction (*p*_(age×diet)_ > 0.05); HDL level increased markedly with age, whereas GAA supplementation significantly decreased it. LDL level was not significantly affected by age, dietary GAA or their interaction (*p*_(age)_ > 0.05, *p*_(diet)_ > 0.05, *p*_(age×diet)_ > 0.05).

### 3.5. Antioxidant Capacity

**Figure 5:**
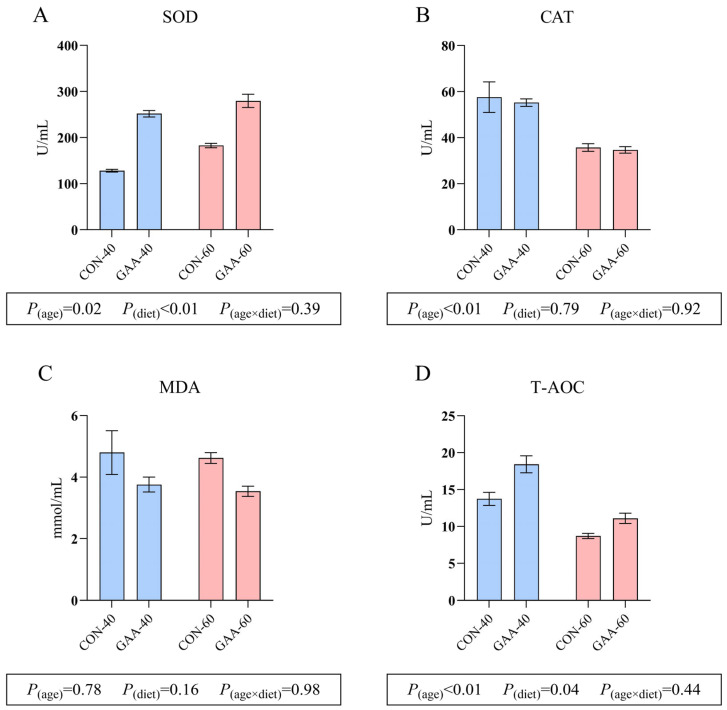
Serum antioxidant parameters of rabbits fed diets with or without GAA. (**A**) Superoxide dismutase (SOD), (**B**) Catalase (CAT), (**C**) Malondialdehyde (MDA), (**D**) Total antioxidant capacity (T-AOC). The error bar represents the standard error of means (SEM). *n* = 3.

As shown in Figure 5, SOD activity was significantly influenced by age (*p*_(age)_ < 0.05) and highly significantly influenced by GAA (*p*_(diet)_ < 0.01), without a significant age × diet interaction (*p*_(age×diet)_ > 0.05); SOD activity increased significantly with advancing age, and GAA supplementation significantly upregulated it. CAT activity was significantly affected only by age (*p*_(age)_ < 0.01), decreasing markedly as age increased, whereas dietary GAA and the age × diet interaction had no significant effect (*p*_(diet)_ > 0.05, *p*_(age×diet)_ > 0.05). MDA concentration was not significantly affected by age, dietary GAA, or their interaction (*p*_(age)_ > 0.05, *p*_(diet)_ > 0.05, *p*_(age×diet)_ > 0.05). T-AOC was significantly influenced by age (*p*_(age)_ < 0.01) and dietary GAA (*p*_(diet)_ < 0.05), with no significant interaction (*p*_(age×diet)_ > 0.05); T-AOC decreased markedly with age, while GAA supplementation significantly increased it.

### 3.6. Fatty Acid Metabolism Related Gene Expression

**Figure 6:**
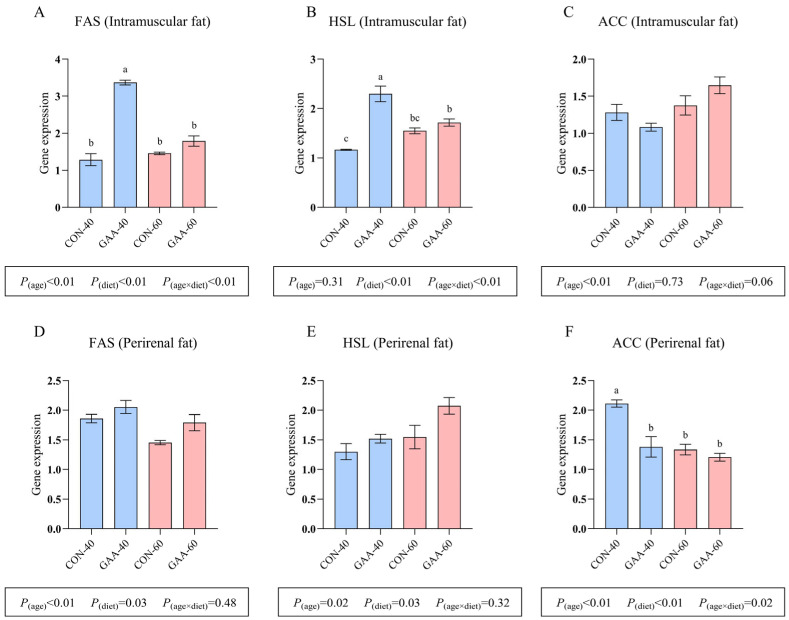
Expression of fatty acid metabolism-related genes in intramuscular and perirenal fat of rabbits fed diets with or without GAA. (**A**,**D**) *FAS*, (**B**,**E**) *HSL*, (**C**,**F**) *ACC*. The error bar represents the standard error of means (SEM). *n* = 3. *FAS*: Fatty acid synthase; *HSL*: Hormone-sensitive lipase; *ACC*: Acetyl coenzyme A carboxylase. Different letter means with different superscript within a bar differ significantly (*p* ≤ 0.05).

As can be seen from Figure 6, in intramuscular fat, the expression of *FAS* was significantly affected by age, dietary GAA, and their interaction (*p*_(age)_ < 0.01, *p*_(diet)_ < 0.01, *p*_(age×diet)_ < 0.01); GAA upregulated *FAS* expression, and the magnitude of upregulation was greater at 40-day-old group. For *HSL*, the main effect of age was not significant (*p*_(age)_ > 0.05), whereas dietary GAA and the age × diet interaction had highly significant effects (*p*_(diet)_ < 0.01, *p*_(age×diet)_ < 0.01); GAA significantly upregulated *HSL* expression, and the regulatory effect differed between ages. *ACC* expression was significantly influenced only by age (*p*_(age)_ < 0.01), with an increasing trend as age advanced, whereas GAA and its interaction with age showed no significant effects (*p*_(diet)_ > 0.05, *p*_(age×diet)_ > 0.05).

In perirenal fat, both age and dietary GAA had significant main effects on *FAS* and *HSL* expression (*p*_(age)_ < 0.05, *p*_(diet)_ < 0.05), without significant age × diet interactions (*p*_(age×diet)_ > 0.05). Increasing age downregulated *FAS* and upregulated *HSL*, while GAA supplementation significantly upregulated the expression of both genes, indicating an independent mode of regulation. For *ACC* in perirenal fat, age, dietary GAA, and their interaction were all significant (*p*_(age)_ < 0.01, *p*_(diet)_ < 0.01, *p*_(age×diet)_ < 0.05); both increasing age and GAA addition significantly suppressed *ACC* expression, and a clear interactive regulatory effect was evident.

## 4. Discussion

Meat rabbits are generally ready for slaughter at around 80 days of age. Accordingly, 40-day-old rabbits were used in the present experiment, which reached the slaughter weight after a 45-day feeding period. However, given that consumers in some areas of Fujian province prefer larger rabbits, 60-day-old rabbits were additionally included to complement the trial. This experimental design also aimed to provide data to support the identification of the optimal period for dietary GAA supplementation in meat rabbits.

As the sole direct metabolic precursor of creatine, dietary GAA supplementation has been shown to increase endogenous creatine levels, thereby enhancing energy metabolism in animals [18]. However, the effects of GAA on growth performance remain inconsistent across published studies: dietary GAA inclusion was reported to improve growth performance and feed utilization efficiency in broilers [19] and in swine from weaning to finishing [13]. In contrast, other studies observed no significant growth response to GAA in chickens [20,21] or finishing pigs [22]. In rabbits, research on GAA application is still limited, with one previous study reporting improved weight gain in growing rabbits fed 0.04–0.12% GAA [23], which contrasts with the present findings that dietary supplementation with 100 mg/kg GAA failed to induce significant changes in the growth and carcass performance of rabbits. These discrepancies may be attributed to differences in animal species, age, basal diet composition, GAA dosage, or experimental conditions, warranting further investigation. Beyond growth performance, organ indices provide critical insight into the physiological status and functional load of key organs in response to dietary interventions [24]. Although creatinine and other GAA-derived metabolites are primarily excreted via the kidneys [25], no increased renal functional burden was observed in this study. Furthermore, the absence of significant changes in heart, liver, spleen and lung indices implies that GAA supplementation did not impose additional metabolic stress on the experimental animals. Additional investigations are required to validate these results and clarify their long-term physiological implications.

Meat quality is primarily assessed by tenderness, water-holding capacity, and pH. Shear force is widely used to assess meat tenderness, while drip loss reflects the water-holding capacity of muscle proteins during processing and storage [26]. Dietary GAA has been shown to modulate meat quality traits across livestock species, with previous work reporting reduced shear force in fattening pigs [12] and bulls [27], as well as improved shear force and muscle fiber cross-sectional area in pigs, accompanied by reduced muscle fiber density [28]. In the present study, GAA supplementation exerted a beneficial effect on meat tenderness, which was confirmed by the lowered shear force and decreased muscle fiber density. These findings are consistent with previous reports linking GAA to improved tenderness and altered muscle fiber characteristics [12,27,28]. Muscle fiber properties are closely related to meat tenderness, as muscle fibers account for around 90% of skeletal muscle, leaving the remaining 10% to connective and adipose components [29,30]. Therefore, the observed reduction in muscle fiber density may contribute to the improved tenderness. Furthermore, the effect of GAA on shear force was age-dependent, being more pronounced in older rabbits. No significant changes were observed in pH or drip loss following GAA supplementation, which aligns with findings in pigs [28]. A possible explanation involves the role of GAA in energy metabolism. As a creatine precursor, GAA increases muscle creatine and phosphocreatine stores, providing a larger energy reserve that may reduce the reliance on glycogenolysis during post-mortem metabolism [17]. This could limit lactic acid accumulation and help stabilize pH, thereby preserving water-holding capacity. However, the exact mechanisms linking GAA to muscle fiber remodeling and tenderness, as well as the age-dependent responses, warrant further investigation.

Serum ALT and AST are widely used biomarkers of hepatocellular integrity, with elevated levels typically indicating hepatocyte membrane damage or impaired liver function [31]. In the present study, two-way ANOVA revealed that ALT and AST activities were significantly influenced only by age (*p* < 0.01), with ALT decreasing and AST increasing as age advanced, while neither dietary GAA nor the age × diet interaction had a significant effect (*p* > 0.05). The age-dependent increase in AST may reflect normal physiological maturation of cardiac and skeletal muscle, where AST is abundant. The absence of a GAA effect on these liver enzymes suggests that in the context of this trial, dietary addition of GAA failed to exert a measurable hepatoprotective or hepatotoxic effect in rabbits. Regarding lipid metabolism, HDL is responsible for transporting cholesterol retrogradely from peripheral tissues to the liver, while LDL is associated with increased cardiovascular risk [32]. HDL levels showed significant variations in response to age (*p* < 0.01) and dietary GAA (*p* < 0.05), without a significant interaction. Although HDL increased with age, GAA supplementation significantly reduced serum HDL levels in both age groups, while LDL remained unaffected. This finding is in line with a previous study conducted in broilers receiving dietary supplementation of 1200 mg/kg GAA. [33]. The reduction in HDL by GAA suggests a potential regulatory role of GAA in lipid metabolism, possibly influencing cholesterol transport and redistribution. The physiological implications of this HDL-lowering effect, particularly in the context of long-term health and meat quality, merit further investigation.

The antioxidant defense system, which consists of enzymatic antioxidants including SOD and CAT as well as non-enzymatic antioxidants, exerts a pivotal role in protecting cells from oxidative damage, while MDA is widely recognized as a core biomarker of lipid peroxidation [34]. In the present study, GAA supplementation significantly enhanced serum antioxidant capacity, as evidenced by elevated SOD activity and T-AOC. These findings are consistent with a study in broilers, in which GAA has been reported to decrease reactive oxygen species (ROS) and MDA levels and elevate T-AOC [35]. Similarly, prior investigations revealed that GAA can enhance hepatic SOD and glutathione peroxidase (GSH-Px) activities, accompanied by a reduction in MDA levels in tilapia [36]. Collectively, these results indicate that GAA enhances systemic antioxidant defense, which may contribute to improved meat quality by reducing oxidative stress-induced damage.

FAS, HSL, and ACC are key enzymes regulating lipid metabolism. FAS acts as a key enzyme driving the biosynthesis of long-chain saturated fatty acids, with its expression level positively linked to fat deposition in animals [37,38,39]. HSL hydrolyzes triglycerides into free fatty acids and glycerol, acting as a core mediator of lipolysis [40,41]. In de novo fatty acid synthesis, ACC acts as the major rate-limiting enzyme, providing malonyl-CoA for subsequent chain elongation by FAS [42]. Dietary GAA has been shown to improve carcass quality by reducing excessive fat deposition in livestock [9]. In this trial, GAA upregulated *FAS* and *HSL* expression in the intramuscular fat and perirenal fat, suggesting a balanced regulation of lipid synthesis and hydrolysis that may help prevent excessive fat accumulation. Notably, GAA exhibited age-dependent regulation of intramuscular *FAS* and *HSL* expression, being more pronounced in younger rabbits. In contrast, dietary supplementation with GAA downregulated *ACC* expression in perirenal fat in an age-dependent manner, with a more pronounced effect observed in younger rabbits, indicating reduced de novo lipogenic capacity in this depot. This tissue-specific response may reflect a metabolic adaptation to energy demands: under conditions of enhanced energy turnover, suppression of ACC could shift metabolism toward fatty acid oxidation, thereby supporting energy homeostasis [9]. These observations offer a novel perspective on the modulatory action of GAA in lipid metabolism, although the long-term implications for fat deposition and meat quality warrant further investigation.

Collectively, the findings of this study demonstrate that dietary GAA supplementation exerts multiple beneficial effects on rabbit meat quality, antioxidant capacity, and lipid metabolism, despite having no significant impact on growth performance. Dietary supplementation with GAA improved meat tenderness in rabbits, together with enhanced systemic antioxidant defense and modulated expression of lipid metabolism-related genes. This suggests that GAA acts through integrated mechanisms involving energy metabolism, oxidative stress reduction, and tissue-specific regulation of fat deposition. These effects are age-dependent, highlighting the complexity of the biological functions of GAA in rabbits. While the present study provides a comprehensive evaluation of GAA in rabbits, further in-depth investigations are still required to decipher the molecular pathways underlying these observations, particularly the crosstalk between muscle and adipose tissues. Additionally, long-term studies assessing the persistence of these effects and their translation to consumer-relevant outcomes, such as sensory attributes and nutritional value of rabbit meat, would be valuable. Meanwhile, this study also has certain limitations. At that time, budget constraints and limited laboratory capacity led to a relatively small sample size, which may have reduced the statistical power of our findings to some extent. In future studies, a larger sample size and a wider range of indicators will be needed to achieve a deeper mechanistic understanding. Furthermore, the present study did not include a detailed economic analysis because the GAA product was provided free of charge by the manufacturer for research purposes, and its commercial price was not available at the time of the study. Once the product is marketed with a defined price, future research under commercial production conditions should incorporate a full cost–benefit analysis to evaluate the economic viability of GAA supplementation for rabbit producers. Overall, GAA represents a promising feed additive for improving the functional quality of rabbit meat, aligning with the growing demand for healthier and sustainably produced animal products.

## 5. Conclusions

In summary, the addition of GAA to the diet did not cause any significant changes in the growth performance and carcass trait profiles of New Zealand white rabbits. However, it improved the tenderness of the longissimus thoracis et lumborum and enhanced systemic antioxidant capacity. Furthermore, GAA altered the expression of pivotal genes linked to fat deposition. Collectively, the results of this work offer a scientific basis for the utilization of GAA as a nutritional strategy to improve meat quality in rabbit production, and offer a reference for the optimal timing of GAA supplementation. Additional systematic in-depth investigations are required to fully decipher the molecular mechanisms underlying these effects.

## Figures and Tables

**Table 1:** Diet composition and nutrient levels (air-dried basis).

Item	Contents, %
Alfalfa hay meal	28.50
Corn	26.20
Rapeseed meal	5.00
Wheat bran	15.40
Soybean meal	8.40
Rice bran	6.00
Squeeze soybeans	5.00
Ca(HCO_3_)_2_	0.70
NaCl	0.50
Methionine	0.10
Lysine	0.20
Premix ^1^	4.00
Total	100.00
Nutrient level ^2^	
Moisture (%)	12.80
Crude Protein (%)	15.48
Crude Fiber (%)	12.25
Ether Extract (%)	3.63
Calcium (%)	0.73
Phosphorus (%)	0.37
Lysine (%)	0.76
Methionine + Cystine (%)	0.45
Digestible energy (MJ/kg)	10.46

^1^ The premix provided the following per kg of diets: Cu 5 mg, Fe·50 mg, Zn 50 mg, Mn 8 mg, Mg 0.03 mg, I 0.5 mg, Se 0.1 mg, Co 0.25 mg, VA 6000 IU, VD_3_ 1000 IU, VK 1.0 IU, VE 50 mg, VB_1_ 1 mg, VB_2_ 3 mg, VB_3_ 1.2 mg, VB_12_ 0.01 mg, folic acid 0.2 mg, pantothenic acid 10 mg, niacin 30.0 mg, biotin 0.08 mg, choline 100 mg; ^2^ Moisture, Crude protein (CP), crude fiber (CF), ether extract (EE), calcium (Ca), phosphorus (P), Lysine (Lys) and Methionine + Cystine (Met + Cys) were measured and recorded. CP was determined by the Kjeldahl method; CF were measured using the filter bag technique; Ca was determined by the ethylenediaminetetraacetic acid (EDTA) complexometric titration method; P was measured by spectrophotometry at a wavelength of 400 nm with the molybdenum blue reaction. Amino Acid was measure by HPLC. The digestive energy is the calculated value.

**Table 2:** Nucleotide sequences of primers used for quantitative real-time PCR assays.

Items	Primer Sequences (5′–3′)
*FAS*	F: GCTGGCTCACTGTCCACAAG
	R: CTGGTTTGCCCTCATTGCTT
*HSL*	F: CTCCTACGACCTGCGTGAAG
	R: CAGCTCTTGAGGTAGGGCTC
*ACC*	F: TGTCCGCACCGACTGTAATC
	R: AGTTGGTGTCAGGCGAATGT
*ACTIN*	F: GTGCTTCTAGGCGGACTGTT
	R: TCGGCCACATTGCAGAACTT

**Table 3:** Growth performance and carcass traits of rabbits in different ages after addition of GAA.

Items	40-Day-Old Group		60-Day-Old Group		*p*-Value		
	CON-40	GAA-40	CON-60	GAA-60	Age	Diet	Age × Diet
Growth performance							
Initial body weight (kg)	1.19 ± 0.03	1.29 ± 0.04	1.82 ± 0.05	1.92 ± 0.06	<0.01	0.10	0.99
Final body weight (kg)	2.40 ± 0.03	2.37 ± 0.07	3.51 ± 0.08	3.62 ± 0.09	<0.01	0.10	0.33
Average daily gain (g)	26.99 ± 0.90	24.08 ± 1.91	27.32 ± 1.33	27.45 ± 1.28	0.19	0.33	0.29
Average daily feed intake (g)	132.43 ± 4.88	125.98 ± 4.04	220.38 ± 13.52	223.21 ± 10.61	<0.01	0.84	0.62
F/G	4.64 ± 0.27	5.29 ± 0.63	8.28 ± 0.37	8.26 ± 0.50	<0.01	0.50	0.47
Carcass traits							
Live weight before slaughter (kg)	2.70 ± 0.04	2.99 ± 0.14	3.86 ± 0.06	3.81 ± 0.08	<0.01	0.19	0.07
Carcass weight (kg)	1.35 ± 0.04	1.43 ± 0.06	1.93 ± 0.04	1.87 ± 0.05	<0.01	0.84	0.17
Dressing percentage (%)	49.99 ± 1.05	47.88 ± 0.66	50.00 ± 0.67	49.08 ± 0.66	0.45	0.07	0.46

Note: F/G, feed/gain ratio. The results are presented as the mean ± SEM. For growth performance, *n* = 10. For carcass traits, *n* = 5.

**Table 4:** Meat quality parameters and muscle fiber characteristics of rabbits fed diets with or without GAA.

Items	40-Day-Old Group		60-Day-Old Group		*p*-Value		
	CON-40	GAA-40	CON-40	GAA-40	Age	Diet	Age × Diet
pH_45min_	6.98 ± 0.07	6.98 ± 0.04	6.58 ± 0.08	6.67 ± 0.06	<0.01	0.49	0.49
Drip loss (%)	2.45 ± 0.16	2.48 ± 0.15	2.82 ± 0.19	2.90 ± 0.21	0.04	0.76	0.89
Shear force (N)	19.24 ± 1.80 ^c^	17.56 ± 1.94 ^c^	37.99 ± 1.67 ^a^	28.56 ± 1.11 ^b^	<0.01	<0.01	0.03
Muscle fiber diameter (mm)	0.05 ± 0.01	0.06 ± 0.01	0.07 ± 0.01	0.07 ± 0.01	0.15	0.62	0.62
Total muscle fiber area (mm^2^)	0.21 ± 0.01	0.18 ± 0.01	0.17 ± 0.01	0.18 ± 0.01	0.06	0.33	0.06
Muscle fiber density (N/mm^2^)	507.70 ± 5.68	468.74 ± 3.84	327.10 ± 9.45	307.32 ± 4.17	<0.01	<0.01	0.14

Note: Data are presented as mean ± SEM, *n* = 5. Different letter means with different superscript within a bar differ significantly (*p* ≤ 0.05).

## Data Availability

The original contributions presented in this study are included in the article or Supplementary Material. Further inquiries can be directed to the corresponding author.

## Supplementary Materials

The following supporting information can be downloaded at: https://www.mdpi.com/article/10.3390/ani16121827/s1, Table S1. Effect of dietary GAA supplement in growing rabbits (preliminary trial).

## Abbreviations

The following abbreviations are used in this manuscript:

ACC	Acetyl-CoA carboxylase
ACTIN	Endogenous reference gene β-actin
ADFI	Average daily feed intake
ADG	Average daily gain
ALT	Alanine aminotransferase
AST	Aspartate aminotransferase
ATP	Adenosine Triphosphate
CAT	Catalase
CF	Crude fiber
CP	Crude protein
EDTA	Ethylenediaminetetraacetic acid
EE	Ether extract
F/G	Feed-to-gain ratio
FAS	Fatty acid synthase
GAA	Guanidinoacetic acid
GSH-Px	Glutathione peroxidase
HDL	High-density lipoprotein
HSL	Hormone-sensitive lipase
LDL	Low-density lipoprotein
MDA	Malondialdehyde
NRC	National Research Council
ROS	Reactive oxygen species
SOD	Superoxide dismutase
T-AOC	Total antioxidant capacity
