# Supplementary material for: Dietary Guanidinoacetic Acid Improves Meat Tenderness and Antioxidant Capacity in Rabbits via Modulating Muscle Fiber Characteristics and Fat Metabolism
Source: Animals (Basel). 2026 Jun 12;16(12):1827. doi: 10.3390/ani16121827 (PMC13296270; doi:10.3390/ani16121827)
Supplement: Supplementary file 1 [file animals-16-01827-s001.zip › animals-4250999-supplementary.pdf]

Items	GAA content (mg/kg)				*p*-value
	0	50	100	150	
**Growth performance**					
**Initial body weight (kg)**	1.23±0.04	1.29±0.04	1.25±0.03	1.18±0.06	0.37
**Final body weight (kg)**	2.25±0.03	2.33±0.05	2.37±0.07	2.22±0.09	0.34
**Average daily gain (g)**	22.67±1.10	23.11±1.21	24.90±0.97	23.11±1.08	0.49
**Average daily feed intake(g)**	132.43±4.88	130.15±6.52	125.98±4.04	126.21±4.57	0.77
**Meat quantity**					
**pH_{45min}**	6.98±0.07	6.98±0.04	6.95±0.07	6.97±0.05	0.98
**Drip loss (%)**	2.44±0.10	2.51±0.16	2.49±0.15	2.45±0.10	0.97
**Shear force (N)**	20.24±0.50[a]	18.56±0.74ab	16.98±0.34b	17.86±0.72ab	0.02

Table S1 Effect of dietary GAA supplement in growing rabbits (preliminary trial)

The results are presented as the mean ± SEM. For growth performance, n = 8. For carcass traits, n = 3. Different letters indicate significant differences ($p < 0.05$).
